# Factors affecting functional disability in patients with non-specific chronic low back pain: a cross-sectional study

**DOI:** 10.3389/fneur.2024.1367400

**Published:** 2024-05-01

**Authors:** Shenyue Zhang, Huan Yang, Beier Luo, Yajun Cheng, Shengbo Niu, Changwei Yang

**Affiliations:** ^1^Department of Biomedical Sciences, Jockey Club College of Veterinary Medicine and Life Sciences, City University of Hong Kong, Kowloon, Hong Kong SAR, China; ^2^Department of Orthopedics, First Affiliated Hospital of the Naval Medical University, Shanghai, China; ^3^Department of Orthopedics, 83^rd^ Army Group Hospital, Xinxiang, Henan, China

**Keywords:** non-specific chronic low back pain, functional disability, patient self-reported outcome, health-related quality of life, sagittal parameters, cross-sectional study

## Abstract

**Background:**

Knowledge about factors affecting functional disability in patients with non-specific chronic low back pain (NSCLBP) is helpful in guiding treatment, but there has been little systematic research on this topic. This study aimed to identify independent factors contributing to functional disability in NSCLBP patients especially the impact of sagittal parameters and body postures in work, learning, and daily life.

**Methods:**

Sociodemographic data, sagittal parameters, Oswestry Disability Index (ODI), Numeric Rating Scale (NRS), and 36-item Short Form Health Survey (SF-36) of NSCLBP patients were collected. Patients were divided into a low-functional disability group (ODI ≤ 20) and a high-functional disability group (ODI > 20), and the ODI was converted to ranked ODI (RODI) accordingly. Sociodemographic data, sagittal parameters, NRS, and SF-36 were compared by univariate analysis between both groups. A correlation analysis of the aforementioned factors with the RODI was conducted. The sociodemographic data and sagittal parameters related to the RODI were analyzed by logistic regression to select potential RODI-associated factors. The level of significance was set at *P* < 0.05.

**Results:**

Age, educational background, daily main posture while working or learning (DMPWL), daily standing time while working or learning (DSTTWL), daily sitting time while resting (DSITR), sacral slope–pelvic tilt (SS-PT), spinosacral angle (SSA), NRS, and SF-36 (except mental health, MH) were different between the two groups (*P* < 0.05). Correlation analysis showed that they were related to the RODI (*P* < 0.05). The logistic regression analysis indicated that the regression coefficients of a college degree, postgraduate diploma, DSITR, and SSA were (*B* = −0.197; *P* = 0.003), (*B* = −0.211; *P* = 0.006), (*B* = −0.139; *P* = 0.039), and (*B* = −0.207; *P* = 0.001), respectively, and the odds ratio (OR) and 95% confidence interval (CI) were 0.489 (0.308; 0.778), 0.299 (0.125; 0.711), 0.875 (0.772; 0.993), and 0.953 (0.925; 0.981), respectively.

**Conclusion:**

Educational background, DSITR, and SSA are independent factors affecting functional disability in NSCLBP patients. NSCLBP patients with a lower educational background, shorter DSITR, or smaller SSA should be taken into account in clinical practice and therapeutic choices. Extending sitting time for rest and the avoidance of a forward-leaning standing position are beneficial for reducing functional disability in NSCLBP.

## Introduction

Non-specific chronic low back pain (NSCLBP) is a musculoskeletal disease with a high incidence among the general population and has a lifetime prevalence in individuals worldwide. The incidence of NSCLBP varies with age, gender, and occupation in individual patients, as well as in different countries and regions. The overall prevalence of NSCLBP among workers in the United States of America is 25.7%, including 24.5% in men, 27.1% in women, 23.8% in younger workers aged 18–40 years, and 27.7% in older workers aged 41–64 years (1). The prevalence in the general population of Sub-Saharan Africa ranges from 18.1 to 28.2% (2) and is 23.4% in Brazilian adults over the age of 20 years (3). However, among primary school teachers in Mekele, Ethiopia, it is as high as 74.8% (4). With undetermined etiology, a high disability rate, and a low cure rate, NSCLBP often results in the work absenteeism of patients, low production efficiency, and a huge economic burden to the patients' families and social healthcare systems (5, 6).

A study of the causes of NSCLBP is helpful for its correct diagnosis, prevention, and treatment. However, multiple factors and the inherent complexity of the pathogenic factors of NSCLBP, coupled with the inconsistent research standards, lead to an uneven level of evidence-based medicine in many research conclusions, and hence the guiding significance for prevention of NSCLBP is limited. As a musculoskeletal disorder associated with disability, the treatment of NSCLBP focuses on reducing pain, disability, and other consequences caused by pain (7). It is suggested that the study on pathogenic factors of NSCLBP is of limited value (8). A scientific classification system has been proposed to classify NSCLBP patients into homogeneous subtypes and provide appropriate treatment strategies (9). The subdivision of the NSCLBP patients reveals that differences in sitting postures are associated with functional disability, which also illustrates the importance of classifying NSCLBP patients (10). The criteria of the US National Institutes of Health (NIH) for NSCLBP research proposes that given the current knowledge, NSCLBP classification based on its impacts is more feasible (7).

At present, research on NSCLBP mainly focuses on risk prediction and evaluation of treatment protocols (11, 12). The common risk factors for NSCLBP include female gender (13), educational background (14), smoking and obesity (15), sedentariness or excessively vigorous physical activity (16, 17), and sitting or standing for more than 2 h (18). Lumbar lordosis (LL) is the pathogenesis of NSCLBP (19, 20). However, whether they are related to functional disability in NSCLBP is undetermined, especially sagittal parameters. Sagittal parameters are associated with the postoperative quality of life in patients with degenerative lumbar scoliosis and adolescent idiopathic scoliosis (21, 22). It was found in our recent previous study that age and spinosacral angle (SSA) were associated with functional disability in NSCLBP patients (23). However, knowing that NSCLBP is a biopsychosocial problem with complex factors affecting its pain and functional disability, it is to be expected that there cannot be a simple relationship between spinal posture in standing and functional disability. We also assumed that functional disability in NSCLBP patients has a certain relationship with body postures in work, learning, and daily life in the modern world. In conclusion, adjusting sociodemographic data and sagittal parameters concurrently is potentially valuable to comprehensively understand the factors affecting functional disability in NSCLBP patients. The combination of sociodemographic data and sagittal parameters may contribute to the new findings. Therefore, this study used sociodemographic data collected at the same time as the previous study (23) to analyze factors affecting functional disability in NSCLBP patients, and factors closely related to working, learning, and lifestyle such as daily main posture while working or learning (DMPWL), daily sitting time while working or learning (DSITWL), daily standing time while working or learning (DSTTWL), daily sitting time while resting (DSITR), and daily standing time while resting (DSTTR) were highlighted and quantified. This is the first study that combined sociodemographic data with sagittal parameters to screen factors affecting functional disability in NSCLBP patients by including as many sagittal parameters as possible while quantifying modern lifestyles.

## Methods

### Participants

The participants of the study were NSCLBP patients who visited the Spine Surgery Outpatient Service of the First Affiliated Hospital of the Naval Military Medical University from February 2021 to August 2021. The study was approved by the Institutional Review Board and Ethics Committee of the said university, and all patients provided informed consent. For each patient, full spine anteroposterior and lateral X-ray radiography was performed by using a vertical 30 × 90 cm film with a constant distance between the subject and the radiographic source. All patients were in a naturally relaxed and comfortable standing posture, with the knee fully extended, the fingers on the clavicle, and the shoulder flexed 45° forward (24). The inclusion and exclusion criteria of the NSCLBP patients are the same as described in our previous article (23). The study was a cross-sectional study reported according to the Strengthening Reporting of Observational Studies in Epidemiology (STROBE) guidelines (25).

### Data collection

**Figure 1:**
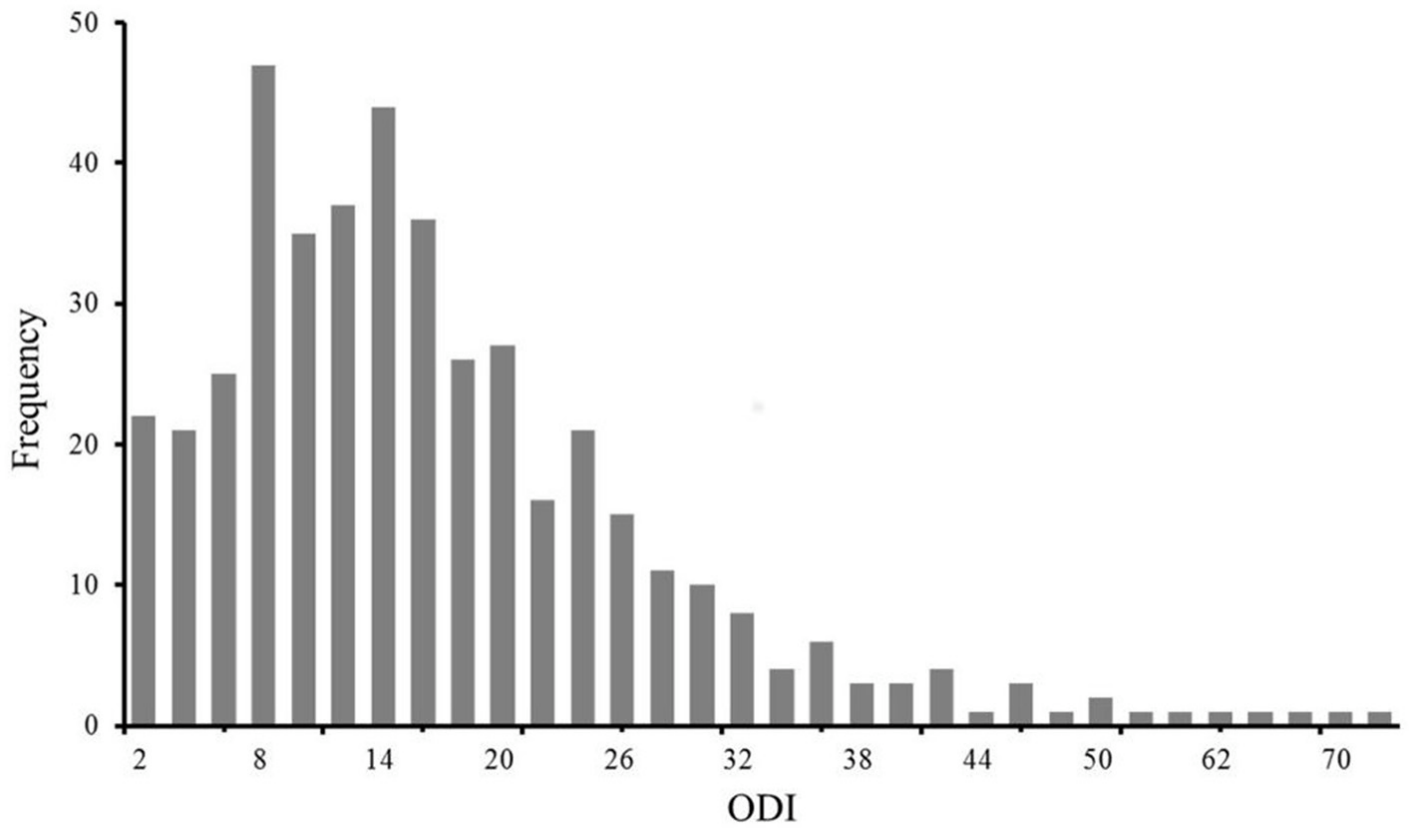
Frequency distribution of the ODI.

The number of participants, patients not eligible for the study and the specific reasons, and the screening process can be referred to in our previous article (23). The final sample size for inclusion was 435 NSCLBP patients. The flow chart of the participants is shown in Figure 1 in our previous study (23). Sociodemographic data, Oswestry Disability Index (ODI), 36-item Short Form Health Survey (SF-36), and Numeric Rating Scale (NRS) were collected by an online questionnaire, in which the sociodemographic data included age, gender, body mass index (BMI), educational background, marriage status, income, smoking, drinking, main nature of work, years of employment, workload, exposure to vibration sources while working, family history of low back pain, DMPWL, DSITWL, DSTTWL, DSITR, and DSTTR. Among the other parameters, DMPWL was derived from the patients' choice of answers to “What is your main posture (standing or sitting) while you are working or learning every day?” DSITWL from the patients' choice of answers to “What is your sitting time while you are working or learning every day?” DSTTWL from the patients' choice of answers to “What is your standing time while you are working or learning every day?” DSITR from the patients' choice of answers to “What is your sitting time while you are resting every day?” and DSTTR from the patients' choice of answers to “What is your standing time while you are resting every day?” The time frame for the answers to the questions ranges from 1 to 10 h.

The ODI was used to assess the functional disability in NSCLBP, the NRS was used to assess pain intensity, and SF-36 was used to assess health-related quality of life (HRQoL). The reliability and validity of simplified Chinese version 2.1 of the ODI make it applicable to Chinese patients (26). SF-36 v2 has also been verified in Chinese patients (27). ODI is the most commonly used indicator to assess acute and chronic low back pain (28, 29). Functional disability was classified into the following five classes: minimal disability (0–20); moderate disability (21–40); severe disability (1–60); crippled (61–80); and being bed-bound (81–100) (28).

The included sagittal parameters were thoracic kyphosis (TK), LL, sacral slope (SS), pelvic incidence (PI), pelvic tilt (PT), sagittal vertical axis (SVA), T1 pelvic angle (TPA), T1 spinopelvic inclination (T1SPi), T9 spinopelvic inclination (T9SPi), spinosacral angle (SSA), sacrofemoral distance (SFD), Barrey ratio, TK/LL, TK-LL, PI-LL, SS/PT, and SS-PT. The measurement methods and measured values of sagittal parameters can be referred to in our previous article (23).

### Statistical analysis

All NSCLBP patients were divided into a low-functional disability group (ODI ≤ 20) and a high-functional disability group (ODI > 20), and the ODI was converted to ranked ODI (RODI) accordingly. The normal distribution was tested by the Shapiro–Wilk test, and the Levene test was used for assessing the homogeneity of variance. Quantitative variables were presented with means and standard deviation (SD) or medians and interquartile (IQR; as appropriate), and qualitative variables were presented with absolute numbers and frequencies (%). The quantitative variables were compared between the two groups by the *t*-test or the rank-sum test. A comparison between the two groups of the unordered qualitative variables was carried out by using the chi-square test or the corrected chi-square test or Fisher's exact test. The Cochran–Mantel–Haenszel test was used to compare the ordered qualitative variables between the two groups. The correlation was analyzed by Spearman's correlation or the chi-square test (the coefficient of contingency was calculated; as appropriate). A logistic regression was conducted to assess the variables associated with the RODI, and the test level for variable inclusion in the equation is 0.05, and the test level for variable exclusion in the equation is 0.1. All statistical analyses were performed using Statistic Package for Social Science 22.0 (SPSS Inc., Chicago, IL). The *p*-value of < 0.05 was considered statistically significant. The evaluation of sample size is mainly based on empirical rules. Multivariate regression analysis generally requires that the number of samples for event outcomes be 5–10 times the number of independent variables (30).

## Results

**Figure 2:**
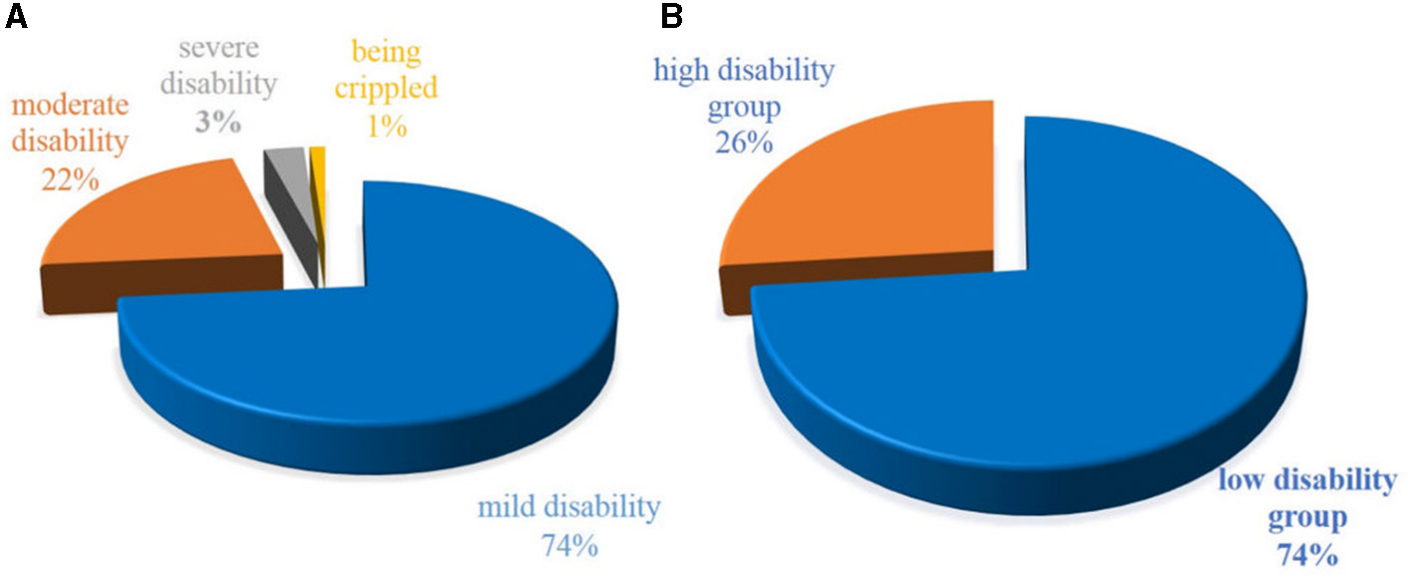
**(A)** The proportion of five classes of functional disability in 435 NSCLBP patients. **(B)** The proportion of the low-functional disability group (ODI ≤ 20) and the high-functional disability group (ODI > 20) in 435 NSCLBP patients.

A total of 435 NSCLBP patients (262,60% were female patients) with a median (IQR) age of 34 (16) years, a median (IQR) BMI of 22.9 (4.4) kg/m^2^, and a median (IQR) ODI of 14 (14) were included in the study (Supplementary Table 1). The frequency distribution of the ODI is shown in Figure 1. According to the five classes of functional disability, 320 (74%) patients had mild disability, 97 (22%) patients had moderate disability, 13 (3%) patients had severe disability, five (1%) patients were crippled, and no patient was bed-bound. Of them, 320 (74%) patients were included in the low-disability group, and 115 (26%) patients were included in the high-disability group (Figure 2). Other characteristics of the 435 NSCLBP patients, their subgroups, and the comparison of all variables between the two subgroups are summarized in Supplementary Table 1.

Age, educational background, DMPWL, DSTTWL, DSITR, SS-PT, SSA, NRS, and SF-36 (except mental health, MH) with statistical differences between the two groups are summarized in Table 1, and the RODI was found to be associated with them (except MH; *P* < 0.05; Table 2). The number of independent variables that can finally be included in the regression equation ranged from 11 to 23. There were seven variables used in this study, which was in line with the empirical rules. The logistic regression analysis indicated that the regression coefficients of a college degree, postgraduate diploma, DSITR, and SSA were (*B* = −0.197; *P* = 0.003), (*B* = −0.211; *P* = 0.006), (*B* = −0.139; *P* = 0.039), and (*B* = −0.207; *P* = 0.001), respectively, and the odds ratio (OR) and 95% confidence interval (CI) were 0.489 (0.308; 0.778), 0.299 (0.125; 0.711), 0.875 (0.772; 0.993), and 0.953 (0.925;0.981), respectively (Table 3).

**Table 1:** Variables with statistical differences between the two subgroups.

**Variables**	**All patients**	**ODI ≤ 20**	**ODI > 20**	***P*-value**
	**(n** = **435)**	**(*****n*** = **320)**	**(*****n*** = **115)**	
Age (years); median (*IQR*)	34 (16)	33 (13)	37 (20)	0.002
Educational background, *n* (%)				0.000
High school or below	179 (41)	116 (36)	63 (55)	
College degree	207 (48)	162 (51)	45 (39)	
Postgraduate diploma	49 (11)	42 (13)	7 (6)	
DMPWL, *n* (%)				0.037
Standing posture	119 (27)	79 (25)	40 (35)	
Sitting posture	316 (73)	241 (75)	75 (65)	
DSTTWL (hours), median (*IQR*)	0 (2)	0 (0)	0 (4)	0.049
DSITR (hours), median (*IQR*)	3 (2)	3 (2)	3 (2)	0.034
SS-PT (°), mean (*SD*)	19.7 (11.9)	20.5 (12.0)	17.2 (11.4)	0.011
SSA (°), mean (SD)	124.5 (7.8)	125.1 (7.7)	122.7 (7.6)	0.004
NRS, median (*IQR*)	3 (3)	3 (2)	4(3)	0.000
SF-36				
PF, median (*IQR*)	80 (30)	85 (20)	65 (25)	0.000
RP, median (*IQR*)	100 (50)	100 (25)	50(100)	0.000
BP, median (*IQR*)	69 (24)	80 (18)	58 (35)	0.000
GH, median (*IQR*)	50 (25)	53 (28)	45 (23)	0.000
VT, median (*IQR*)	65 (30)	70 (25)	60 (25)	0.009
SF, median (*IQR*)	88 (25)	88 (25)	75 (25)	0.000
RE, median (*IQR*)	100 (67)	100 (67)	66 (100)	0.002

BP, bodily pain; DMPWL, daily main posture while working or learning; DSITR, daily sitting time while resting; DSTTWL, daily standing time while working or learning; GH, general health; IQR, interquartile range; NRS, numerical rating scale; ODI, Oswestry Disability Index; PF, physical function; RE, role emotional; RP, role physical; SD, standard deviation; SF, social function; SF-36, Short Form 36 Health Survey; SS-PT, sacral slope–pelvic tilt; SSA, spinosacral angle; VT, vitality.

**Table 2:** Correlations of sociodemographic characteristics, sagittal parameters, NRS, SF-36, and RODI of 435 NSCLBP patients.

Age	0.126^**^	PF	−0.470^**^
Educational background	−0.174^**^	RP	−0.334^**^
DMPWL	0.488^**^	BP	−0.276^**^
DSTTWL	0.094^*^	GH	−0.191^**^
DSITR	−0.102^*^	VT	−0.125^**^
SS-PT	−0.115^*^	SF	−0.241^**^
SSA	−0.116^*^	RE	−0.148^**^
NRS	0.266^**^	MH	−0.064

BP, bodily pain; DMPWL, daily main posture while working or learning; DSITR, daily sitting time while resting; DSTTWL, daily standing time while working or learning; GH, general health; IQR, interquartile range; MH, mental health; NRS, Numerical Rating Scale; ODI, Oswestry Disability Index; PF, physical function; RE, role emotional; RODI, ranked Oswestry Disability Index; RP, role physical; SD, standard deviation; SF, social function; SF-36, Short Form 36 Health Survey; SS-PT, sacral slope-pelvic tilt; SSA, spinosacral angle; VT, vitality.

^*^P < 0.05; ^**^P < 0.01.

**Table 3:** Binary logistic regression analysis of independent factors affecting functional disability in NSCLBP patients.

**Variables**	**B**	**SE**	**Wald**	***P*-value**	**OR**	**95% CI of OR**
Constant	5.829	1.886	9.550	0.002		
Educational background^*^						
College degree	−0.715	0.237	9.122	0.003	0.489	(0.308, 0.778)
postgraduate diploma	−1.208	0.443	7.450	0.006	0.299	(0.125, 0.711)
DSITR	−0.133	0.064	4.256	0.039	0.875	(0.772, 0.993)
SSA	−0.048	0.015	10.461	0.001	0.953	(0.925, 0.981)

B, regression coefficient; CI, confidence interval; DSITR, daily sitting time while resting; NSCLBP, non-specific chronic low back pain; SE, standard error; OR, odds ratio; SSA, spinosacral angle.

^*^Control group was patients with high school education or below.

## Discussion

Unlike the chronic pain symptoms that are usually accompanied with other diseases, NSCLBP is a condition that requires specific treatment and care (31). Conservative therapy is the first-line option for NSCLBP to alleviate pain and improve functional disability, and researching factors affecting functional disability can help medical staff identify patients with severe functional disability and guide the treatment. In our series, 97 (22.30%) patients had moderate disability, 13 (2.99%) patients had severe disability, 5 (1.15%) patients were crippled, and no patient was bed-bound. To satisfy the sample size of statistical analysis, we converted the ODI to RODI. Univariate correlation analysis showed that the RODI was positively correlated with the NRS and negatively correlated with seven dimensions in SF-36 (except MH), indicating that the greater the pain, the more severe the disability, and the worse the quality of life, and the grouping of cases with low and high disability has clinical significance. It has also been shown that age, educational background, DMPWL, DSTTWL, DSITR, SS-PT, and SSA were related to functional disability in NSCLBP, indicating that functional disability was more severe in patients with older age or lower educational background or those with a standing posture as the main daily posture while working or learning, and the disability increased with longer DSTTWL or shorter DSITR. After adjusting for confounding factors in logistic regression analysis, educational background, DSITR, and SSA were found to be independent factors affecting functional disability in NSCLBP patients. Compared with the patients with a high-school or below educational background, the OR for increased disability in NSCLBP patients with a college degree and postgraduate diploma was 0.30-fold and 0.49-fold higher, respectively. The OR was 0.88-fold higher for every 1 h increase in the DSITR and 0.95-fold higher for every 1 degree increase (reduced kyphosis). Educational background, DSITR, and SSA were independent protective factors affecting functional disability in NSCLBP; the higher the educational background, the longer the DSITR, or the greater the SSA (reduced kyphosis), the lower the risk of increased disability. Thus, the findings of the present study may serve as a reminder for clinicians to pay more attention to patients with lower educational backgrounds, shorter DSITR, or smaller SSA in clinical practice and therapeutic choices.

It was found in our study that educational background was negatively correlated with the RODI (*r* = −0.174, *P* < 0.01). This may be related to the low socioeconomic status in patients with a low educational background, and there is a higher proportion of NSCLBP patients with disability in people with a low socioeconomic status (15). At the same time, patients with a high educational background have strong self-care awareness, such as performing regular physical exercise, which reduces the impact of NSCLBP on physiological function (PF), and the RODI had the highest correlation with PF (*r* = −0.470; *P* < 0.01). The guidelines of the National Institute for Health and Care Excellence (NICE) recommend education and self-care for the early treatment of NSCLBP, including advising and educating patients about the nature of pain, not necessary for bed rest during treatment, and encouraging them to remain active and continue their daily activities, including work (32). As expected, a longer DSITR is beneficial for NSCLBP patients. This may be related to the relatively free sitting posture at rest, and the waist muscles are in a relaxed state. A long, flat, or stiff waist increases the risk of severe NSCLBP, which is difficult to explain by other mechanical factors such as muscle strength and lumbar mobility (8). Therefore, extending the sitting time for rest is beneficial for reducing functional disability in NSCLBP patients and may be an important and simple treatment.

Biological factors (such as old age, overweight or obesity, female gender, current smoking, and co-existing chronic diseases), social conditions (such as low educational background, low per capita household income, singlehood, and living in rural areas), and psychological health conditions (such as the presence of depressive symptoms) are associated with a higher prevalence of NSCLBP (3). However, this study found that age, gender, BMI, smoking, DMPWL, DSITWL, and DSTTWL were unexpectedly unrelated to NSCLBP disability. In our recent previous study (23), age was found to be associated with NSCLBP disability, and this may be related to the fact that fewer variables were included, compared to this study. Sedentariness combined with an incorrect posture has been shown to increase the risk of NSCLBP (18). This could be attributed to the relationship between the factors of the onset of NSCLBP and their association with disability is unclear; in other words, it may have to do with the different purpose of this study, and we suggested that the two overlap but may not be identical. Furthermore, previous studies on low back pain have produced a number of controversial results. An epidemiological study reported an association between reduced disc space found on x-rays of people with sedentary occupations and acute low back pain (33, 34); for instance, motor vehicle driving and sedentary occupations were considered to have a relatively higher risk of disc space reduction and acute low back pain, but the authors emphasized that further research was needed to confirm or refute the association of the sitting posture with disc degeneration and acute low back pain (33). However, there was also a study on 45 male monozygotic twin pairs that refuted this association, one in each twin pair spent more than five times as much time driving a motor vehicle during his lifetime as the other, yet there was no difference in lumbar disc degeneration on magnetic resonance imaging (35). Based on the lumbar flexion commonly involved in sitting relative to standing posture (36) and related epidemiological study (35), it was found that lumbar flexion associated with the sitting posture had no more a serious impact on the disc health or the onset of NSCLBP than did a relatively extended standing posture. It may also be related to the small sample size of this study and the uneven proportion of patients with different degrees of disability. Moreover, the sitting posture is not described specifically, such as whether there is rotation or not. However, lumbar rotation in a sitting posture is an important part of daily life and activities of different occupations (such as dentists, cashiers, and laboratory workers).

Sociodemographic data such as educational background and DSITR were not included in our previous study (23), but SSA remained an independent factor after they were included in this study, indicating that SSA is an important factor associated with functional disability in NSCLBP. The cutoff point of SSA was 127.35, which would be important for clinical applicability (23). For its definition, SSA is the combined reflection of the reduction in LL and SS, which is a cumulative gain, and enhances the ability of SSA to distinguish NSCLBP disability (23). SSA can comprehensively reflect the compensatory state of sagittal balance in NSCLBP patients and poor sagittal balance represented by decreased SSA is a risk factor for increased disability in NSCLBP patients (23). The avoidance of body forward leaning in a standing position is beneficial for reducing functional disability in NSCLBP. NSCLBP is a biopsychosocial problem in which the patient's anatomical injury interacts with psychosocial conditions (37). Central pain regulation mechanisms and pain cognition play an important role in the development of persistently disabling NSCLBP (15). Hashmi et al. found that brain activity in patients with acute or subacute low back pain is limited to areas of acute pain, while brain activity in NSCLBP patients is limited to emotional circuits (38). Patients with chronic pain have changes in the regions involved in the emotional and cognitive regulation of pain in the brain (39), which may explain why patients with persistent pain are prone to developing depression and anxiety (40). One research has highlighted emotional distress as a factor that potentially increases the risk of sustained disability in NSCLBP (15). Emotional distress is an important issue in the management of NSCLBP. However, little is known about how emotional distress occurs and develops in NSCLBP patients. Previous studies showed that factors affecting the onset of NSCLBP included the degree of pain, mental factors, sleep, and quality of life (41, 42). These factors as characteristics of NSCLBP contribute to its diagnosis, but some of these factors interact with NSCLBP (39, 40), and some result from pain and ineffective treatment of NSCLBP (8). Furthermore, the sensitivity of factors with low influence may be reduced by factors with high influence (such as mental factors and physical function), thus it is not scientific and reasonable to study them as pathogenic factors. Therefore, mental and sleep factors as well as related patient self-reported outcomes (PROs) such as physical function, role physical, and mental health were not included as factors affecting functional disability in NSCLBP. In addition, a comprehensive assessment of functional disability in NSCLBP patients should include objective biomechanical and kinematic data such as muscle endurance and strength (43) in addition to PROs.

## Strengths and limitations

To the best of our knowledge, the present study is the first to identify independent factors affecting NSCLBP functional disability by combining sociodemographic data and sagittal parameters. Nevertheless, this study presents several limitations. The first limitation is that the subjects in this study are NSCLBP patients from the hospital, and an uneven proportion of patients with different degrees of disability may not have been fully representative of the general NSCLBP patients. The second limitation is that all variables were collected from PROs, which may lead to subjective results, and the inclusion of objective measurement would have been desirable. The third limitation is that relying on smartphone electronic questionnaires may also lead to selection bias; for instance, patients who were able to complete questionnaires using smartphones may be more educated than those who were unable to complete questionnaires using smartphones, especially older people. However, the questionnaire survey for this study was conducted by a spine surgeon who specifically assisted patients who could not use smartphones to complete the questionnaire to reduce the bias caused by survey methods. Finally, the cross-sectional study did not allow us to establish causality between independent factors and functional disability in NSCLBP patients.

## Conclusion

Educational background, DSITR, and SSA are independent factors affecting functional disability in NSCLBP patients. Functional disability is severer in patients with a lower educational background, shorter DSITR, or smaller SSA. NSCLBP patients with a lower educational background, shorter DSITR, or smaller SSA should be taken into account in clinical practice and therapeutic choices. Extending sitting time while resting and avoidance of the body forward leaning while standing are beneficial for reducing functional disability in NSCLBP.

## Author's note

It was found in our recent previous study that age and spinosacral angle (SSA) were associated with functional disability in NSCLBP patients (23).

## Data availability statement

The original contributions presented in the study are included in the article/Supplementary material, further inquiries can be directed to the corresponding authors.

## Ethics statement

The studies involving humans were approved by the Ethics Committee of The First Affiliated Hospital, Naval Medical University. The studies were conducted in accordance with the local legislation and institutional requirements. The participants provided their written informed consent to participate in this study. Written informed consent was obtained from the individual(s) for the publication of any potentially identifiable images or data included in this article.

## Author contributions

SZ: Writing—original draft, Writing—review & editing. HY: Data curation, Formal analysis, Investigation, Writing—review & editing. BL: Data curation, Formal analysis, Investigation, Writing—review & editing. YC: Data curation, Formal analysis, Investigation, Writing—review & editing. SN: Project administration, Supervision, Validation, Visualization, Writing—review & editing. CY: Conceptualization, Writing—review & editing.
