# Supplementary material for: Factors affecting functional disability in patients with non-specific chronic low back pain: a cross-sectional study
Source: Front Neurol. 2024 May 1;15:1367400. doi: 10.3389/fneur.2024.1367400 (PMC11094354; doi:10.3389/fneur.2024.1367400)
Supplement: Supplementary file 1 [file Table_1.docx]

Supplementary Material

**Factors of functional disability in patients with nonspecific chronic low back pain: a cross-sectional study**

Shenyue Zhang, Huan Yang, Beier Luo, Yajun Cheng, Shengbo Niu^*^, Changwei Yang^*^

*** Correspondence:** Shengbo Niu, Changwei Yang were corresponding authors, E-mail: niushengbo@163.com and changwei_y@qq.com.

# Supplementary Tables

| **TABLE 1** The characteristics of 435 NSCLBP patients, their subgroups and comparison of variables between the two subgroups. | | | | |
| --- | --- | --- | --- | --- |
| Variables | All patients  (n=435) | ODI≤20  (n=320) | ODI＞20  (n=115) | *P* value |
| Age ( years), median ( *IQR* ) | 34 (16) | 33 (13) | 37 (20) | 0.002 |
| Sex, n (%) |  |  |  |  |
| Male | 173 (40) | 133 (42) | 40 (35) | 0.203 |
| Female | 262 (60) | 187 (58) | 75 (65) |  |
| BMI (kg/m^2^) | 22.66 (4.79) | 22.59 (4.70) | 22.99 (5.08) | 0.873 |
| mean (*SD*) | — | — | 23.1 (3.2) |  |
| median ( *IQR* ) | 22.9 (4.4) | 22.3 (4.8) | — |  |
| Educational background, n (%) |  |  |  | 0.000 |
| High school or below | 179 (41) | 116 (36) | 63 (55) |  |
| College degree | 207 (48) | 162 (51) | 45 (39) |  |
| Postgraduate diploma | 49 (11) | 42 (13) | 7 (6) |  |
| Marriage status, n (%) |  |  |  | 0.441 |
| Married | 275 (63) | 200 (63) | 75 (65) |  |
| Widowed | 6 (2) | 3 (1) | 3 (3) |  |
| Divorced | 13 (3) | 10 (3) | 3 (3) |  |
| Unmarried | 141 (32) | 107 (33) | 34 (29) |  |
| Income, n (%) |  |  |  | 0.259 |
| Lower level | 172 (39) | 120 (37) | 52 (45) |  |
| Medium level | 256 (59) | 196 (61) | 60 (52) |  |
| Higher level | 7 (2) | 4 (2) | 3 (3) |  |
| Smoking, n (%) |  |  |  | 0.830 |
| No | 368 (85) | 270 (84) | 98 (85) |  |
| Yes | 67 (15) | 50 (16) | 17 (15) |  |
| Drinking, n (%) |  |  |  | 0.891 |
| No | 289 (66) | 212 (66) | 77 (67) |  |
| Yes | 146 (34) | 108 (34) | 38 (33) |  |
| Main nature of work, n (%) |  |  |  | 0.078 |
| Brain | 216 (50) | 169 (53) | 47 (41) |  |
| Brain & manual | 86 (20) | 61 (19) | 25 (22) |  |
| Manual | 133 (30) | 90 (28) | 43 (37) |  |
| Years of employment, median ( *IQR* ) | 8 (11) | 8 (10) | 9 (14) | 0.537 |
| Work load, n (%) |  |  |  | 0.149 |
| No | 328 (75) | 247 (77) | 81 (70) |  |
| Yes | 107 (25) | 73 (23) | 34 (30) |  |
| Vibration, n (%) |  |  |  | 0.971 |
| No | 390 (90) | 287 (90) | 103 (90) |  |
| Yes | 45 (10) | 33 (10) | 12 (10) |  |
| Family history of low back pain, n (%) |  |  |  | 0.707 |
| No | 316 (73) | 234 (73) | 82 (71) |  |
| Yes | 119 (27) | 86 (27) | 33 (29) |  |
| DMPWL, n (%) |  |  |  | 0.037 |
| Standing posture | 119 (27) | 79 (25) | 40 (35) |  |
| Sitting posture | 316 (73) | 241 (75) | 75 (65) |  |
| DSITWL (hours), median ( *IQR* ) | 6 (8) | 6 (7) | 5 (8) | 0.303 |
| DSTTWL (hours), median ( *IQR* ) | 0 (2) | 0 (0) | 0 (4) | 0.049 |
| DSITR (hours), median ( *IQR* ) | 3 (2) | 3 (2) | 3 (2) | 0.034 |
| DSTTR (hours), median ( *IQR* ) | 2 (2) | 2 (2) | 2 (2) | 0.971 |
| TK (°) |  |  |  | 0.678 |
| Mean (*SD*) | 36.5 (9.9) | 36.5 (9.1) | — |  |
| Median (*IQR*) | — | — | 36.6 (17) |  |
| LL (°) |  |  |  | 0.051 |
| Mean (*SD*) | — | 47.1 (10.2) | 44.9 (11.1) |  |
| Median (*IQR*) | 47.3 (13) | — | — |  |
| TK-LL (°) |  |  |  | 0.101 |
| Mean (*SD*) | -10.0 (10.8) | -10.7 (10.7) | — |  |
| Median (*IQR*) | — | — | -7.6 (15.1) |  |
| TK/LL, Median (*IQR*) | 0.8 (0.2) | 0.8 (0.2) | 0.8 (0.3) | 0.158 |
| PI (°), Median (*IQR*) | 46 (14.1) | 46 (13.2) | 46 (15.6) | 0.583 |
| PI-LL (°) |  |  |  | 0.218 |
| Mean (*SD*) | — | — | 0.9 (10.4) |  |
| Median (*IQR*) | -0.4 (14) | -0.9 (14) | — |  |
| SS (°) |  |  |  | 0.054 |
| Mean (*SD*) | — | — | 31.5 (7.6) |  |
| Median (*IQR*) | 33.3 (9.1) | 33.6 (9.2) | — |  |
| PT (°), Mean (*SD*) | 13.4 (8.1) | 13.1 (8.3) | 14.3 (7.4) | 0.175 |
| SS-PT (°), Mean (*SD*) | 19.7 (11.9) | 20.5 (12.0) | 17.2 (11.4) | 0.011 |
| SS/PT, Median (*IQR*) | 2.3 (2.3) | 2.4 (2.5) | 2.1 (2.3) | 0.448 |
| SVA (mm) |  |  |  | 0.112 |
| Mean (*SD*) | — | -3.0 (26.5) | 1.5 (25.4) |  |
| Median (*IQR*) | -3.4 (33.7) | — | — |  |
| TPA (°), Median (*IQR*) | -7.8 (8.3) | -7.4 (7.8) | -9.1 (9.3) | 0.186 |
| SSA (°), Mean (SD) | 124.5 (7.8) | 125.1 (7.7) | 122.7 (7.6) | 0.004 |
| T1SPi (°), Mean (*SD*) | -4.2 (2.5) | -4.2 (2.5) | -4.1 (2.4) | 0.733 |
| T9SPi (°), Mean (*SD*) | -9.1 (3.3) | -9.0 (3.3) | -9.5 (3.2) | 0.138 |
| Barrey ratio, Median (*IQR*) | -0.07 (1.0) | -0.1 (1.0) | 0.05 (1.0) | 0.070 |
| ODI, Median (*IQR*) | 14 (14) | 12 (8) | 28 (12) | — |
| NRS, Median (*IQR*) | 3 (3) | 3 (2) | 4(3) | 0.000 |
| SF-36 |  |  |  |  |
| PF, Median (*IQR*) | 80 (30) | 85 (20) | 65 (25) | 0.000 |
| RP, Median (*IQR*) | 100 (50) | 100 (25) | 50(100) | 0.000 |
| BP, Median (*IQR*) | 69 (24) | 80 (18) | 58 (35) | 0.000 |
| GH, Median (*IQR*) | 50 (25) | 53 (28) | 45 (23) | 0.000 |
| VT, Median (*IQR*) | 65 (30) | 70 (25) | 60 (25) | 0.009 |
| SF, Median (*IQR*) | 88 (25) | 88 (25) | 75 (25) | 0.000 |
| RE, Median (*IQR*) | 100 (67) | 100 (67) | 66 (100) | 0.002 |
| MH, Median (*IQR*) | 68 (24) | 68 (24) | 64 (20) | 0.184 |
| BMI, Body mass index; BP, bodily pain; DMPWL, Daily main posture while working or learning; DSITR, Daily sitting time while resting; DSITWL, Daily sitting time while working or learning; DSTTR, Daily standing time while resting; DSTTWL, Daily standing time while working or learning; GH, General health; IQR, Interquartile range; LL, Lumbar lordosis; MH, Mental health; NRS, Numerical rating scale; NSCLBP, Nonspecific chronic low back pain; ODI, Oswestry Disability Index; PF, Physical function; PI, Pelvic incidence; PI-LL, Pelvic incidence-lumbar lordosis; PT, Pelvic tilt; RE, Role emotional; RP, Role physical; SD, Standard deviation; SF, Social function; SF-36, Short Form 36 Health Survey; SS, Sacral slope; SS-PT, Sacral slope-pelvic tilt; SS/PT, Sacral slope/pelvic tilt; SSA, Spino-sacral angle; SVA, Sagittal vertical axis; TK, Thoracic kyphosis; TK-LL, Thoracic kyphosis-lumbar lordosis; TK/LL, Thoracic kyphosis/lumbar lordosis; TPA, T1 pelvic angle; T1SPi, T1 spino-pelvic inclination; T9SPi, T9 spino-pelvic inclination; VT, Vitality. | | | | |
